# Prevalence and factors associated with physical function among patients undergoing maintenance hemodialysis in Buraydah, Saudi Arabia: a cross-sectional study

**DOI:** 10.3389/fneph.2026.1935072

**Published:** 2026-10-14

**Authors:** Shereen Ahmed A. Qalawa, Wafaa Abdullah AlQassem, Kholoud Metab Alomeri, Khalid Saad Alhumidi Alharbi, Ahmed Ibrahim ALsallom, Fatma Mohamed Elmansy, Mohamed Goda Elbqry

**Affiliations:** 1 Department of Medical-Surgical Nursing, College of Nursing, Qassim University, Buraydah, Saudi Arabia; 2 Department of Medical-Surgical Nursing, Faculty of Nursing, Port Said University, Port, Said, Egypt; 3 Privatization Affairs Office, Qassim Region, Ministry of Health, Buraydah, Saudi Arabia; 4 King Fahd Specialist Hospital, Qassim Regional, Peritoneal Dialysis Center and Hemodialysis Center, Buraydah, Ministry of Health, Buraydah, Saudi Arabia; 5 Department of Nursing, Medical City, Qassim University, Buraydah, Saudi Arabia; 6 Department of Medical-Surgical Nursing, Faculty of Nursing, Suez Canal University, Ismailia, Egypt

**Keywords:** activities of daily living, chronic kidney disease, end-stage renal disease, maintenance hemodialysis, physical function, Saudi Arabia

## Abstract

**Background:**

Physical function is a key indicator of health status and quality of life among patients undergoing maintenance hemodialysis. Functional impairment is common in this population owing to the combined effects of chronic kidney disease, treatment-related burden, and multiple comorbidities, resulting in reduced independence and poorer health outcomes. However, evidence regarding the prevalence and factors associated with impaired physical function among maintenance hemodialysis patients in Saudi Arabia remains limited.

**Objective:**

This study aimed to determine the prevalence of physical function impairment and examine the factors associated with physical function among patients undergoing maintenance hemodialysis in Buraydah, Saudi Arabia.

**Methods:**

A hospital-based cross-sectional study was conducted at the Dialysis Center of King Fahd Specialized Hospital, Buraydah, Saudi Arabia. A convenience sample of 110 adult patients undergoing maintenance hemodialysis was recruited. Data were collected using a structured, validated questionnaire assessing sociodemographic and clinical characteristics, physical function, activities of daily living, anthropometric measurements, and laboratory parameters. Descriptive statistics and Chi-square tests were used to examine the associations between variables.

**Results:**

Among the 110 participants, 73.6% demonstrated impaired physical function. Age was the only participant characteristic significantly associated with physical function (p = 0.002), whereas no significant associations were observed with sex, marital status, educational level, employment status, dialysis duration, dialysis frequency, family history of kidney disease, or comorbidities. Participants reported substantial limitations in instrumental activities of daily living, particularly cleaning floors, washing clothes, cooking, carrying objects, and engaging in physical exercise, while most remained independent in basic self-care activities.

**Conclusion:**

Impaired physical function is highly prevalent among patients undergoing maintenance hemodialysis, with older age being significantly associated with poorer physical function. Routine assessment of physical function should be incorporated into dialysis care to facilitate the early identification of at-risk patients and support individualized interventions aimed at maintaining functional independence and improving quality of life.

## Introduction

Maintenance hemodialysis is the most commonly used renal replacement therapy for patients with end-stage renal disease (ESRD), substantially improving survival while imposing significant physical, psychological, social, and economic burdens (1). Patients receiving maintenance hemodialysis must adhere to lifelong treatment schedules, dietary and fluid restrictions, medication regimens, and frequent healthcare visits, all of which may adversely affect their independence, daily functioning, and quality of life (2).

The global burden of chronic kidney disease continues to rise, resulting in an increasing number of individuals requiring long-term dialysis (3). This trend poses major challenges to healthcare systems because of escalating treatment costs, frequent hospitalizations, and the need for comprehensive multidisciplinary care (4). Consequently, maintaining physical function has become a key therapeutic objective, as it directly influences patients’ ability to remain independent, participate in daily activities, and achieve better clinical outcomes (2).

Physical function represents an essential component of health status in patients undergoing maintenance hemodialysis. It encompasses the capacity to perform activities of daily living, maintain mobility, and engage in social, occupational, and family roles (5). However, functional performance often deteriorates because of the combined effects of muscle wasting, fatigue, anemia, chronic inflammation, cardiovascular disease, reduced exercise tolerance, pain, sleep disturbances, and multiple comorbidities (6). Declining physical function has been consistently associated with frailty, reduced quality of life, increased hospitalization, greater dependence on caregivers, and higher mortality among patients with ESRD (7).

Previous research has identified several demographic and clinical factors associated with impaired physical function, including older age, prolonged dialysis duration, diabetes mellitus, cardiovascular disease, poor nutritional status, obesity, anemia, and low levels of physical activity (8). Nevertheless, the magnitude and relative contribution of these determinants vary considerably across populations because of differences in healthcare delivery, cultural context, socioeconomic conditions, and disease characteristics. Therefore, locally generated evidence is needed to better understand factors influencing physical function in specific healthcare settings (9).

This study is conceptually guided by Lazarus and Folkman’s Stress and Coping Theory (**Figure 1**), which views chronic illness as a dynamic process in which individuals continuously appraise disease-related demands and mobilize coping resources to adapt to ongoing stressors (10). Maintenance hemodialysis represents a persistent physiological and psychosocial stressor that requires sustained behavioral, emotional, and physical adaptation. According to this framework, patients’ functional status is influenced not only by the severity of their clinical condition but also by the balance between treatment-related demands, personal coping resources, social support, and healthcare interventions (11). From a nursing perspective, this theoretical framework provides a valuable basis for understanding functional decline and supports interventions aimed at strengthening adaptive capacity, promoting self-management, and preserving independence among patients receiving maintenance hemodialysis (12).

**Figure 1:**
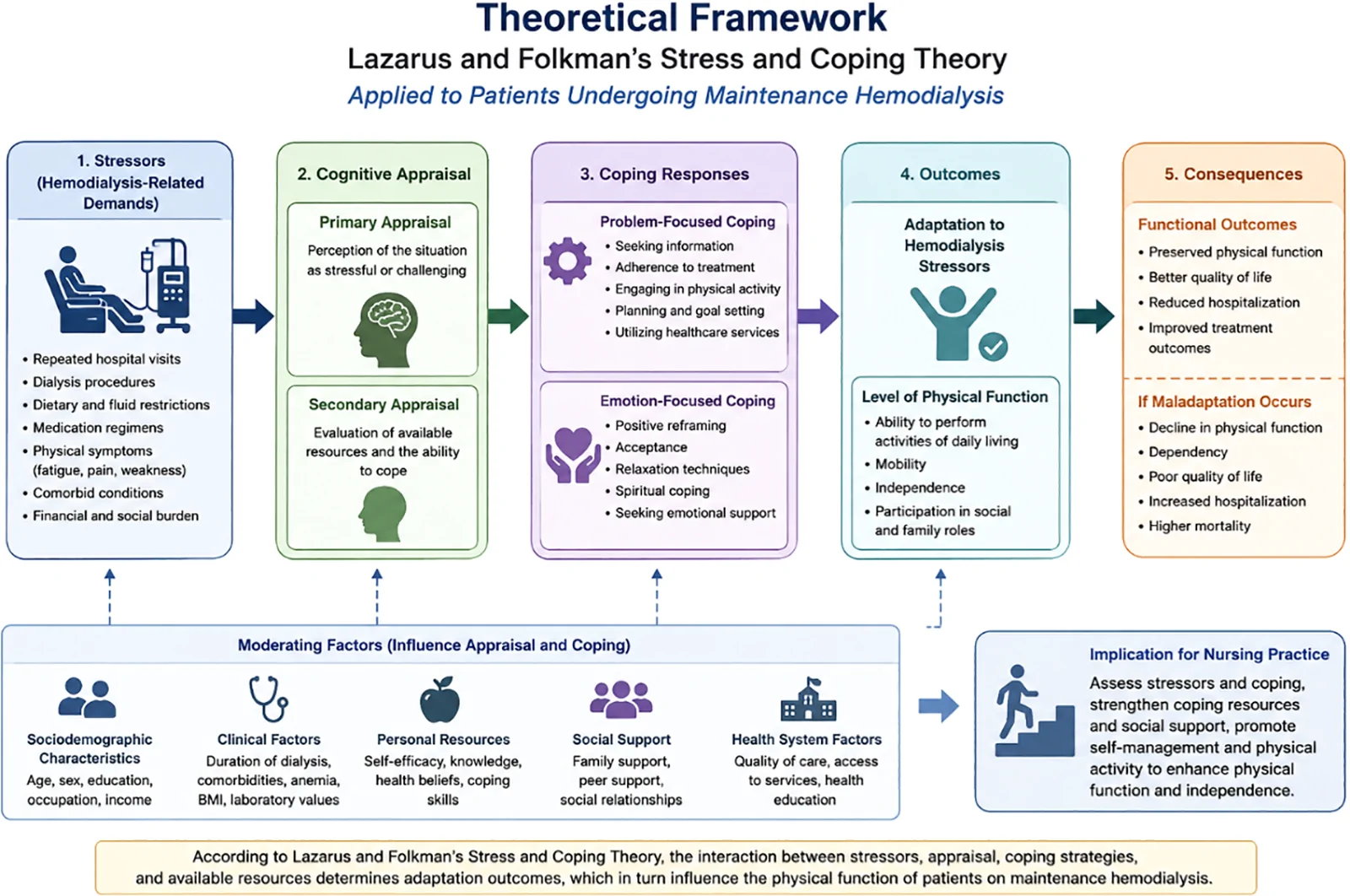
Conceptual framework based on Lazarus and Folkman’s Stress and Coping Theory.

Although numerous studies have investigated quality of life, symptom burden, depression, anxiety, and psychological well-being among patients undergoing maintenance hemodialysis, comparatively fewer studies have specifically examined physical function as a primary outcome, particularly in Middle Eastern populations (13). Moreover, studies that have evaluated physical function have frequently focused on individual clinical predictors or exercise interventions, with limited attention to the combined influence of sociodemographic and clinical characteristics on patients’ ability to perform activities of daily living (14, 15). In Saudi Arabia, evidence describing the prevalence and determinants of physical function impairment remains scarce, especially in the Qassim region. This lack of local evidence limits the ability of healthcare professionals to identify patients at greatest risk of functional decline and to develop culturally appropriate, evidence-based nursing and rehabilitation programs. Therefore, this study aimed to determine the prevalence of physical function impairment and identify its associated sociodemographic and clinical determinants among patients undergoing maintenance hemodialysis in Buraydah, Saudi Arabia. The findings are expected to provide evidence that supports early functional assessment, individualized nursing care, and multidisciplinary rehabilitation strategies designed to maintain independence and improve health outcomes in this vulnerable population.

### Research objectives

1. Determine the prevalence of physical function impairment among patients undergoing maintenance hemodialysis.
2. Assess patients’ physical function and performance of activities of daily living.
3. Examine the association between sociodemographic and clinical characteristics and physical function among patients undergoing maintenance hemodialysis.

### Research hypotheses

H1: Physical function impairment is prevalent among patients undergoing maintenance hemodialysis.
H2: Sociodemographic and clinical characteristics, particularly older age, dialysis duration, and the presence of comorbidities, are significantly associated with physical function among patients undergoing maintenance hemodialysis.

## Methods

### Study design

A hospital-based analytical cross-sectional study was conducted among adult patients undergoing maintenance hemodialysis at King Fahd Specialized Hospital, Buraydah, Saudi Arabia.

### Study setting

The study was conducted at the Dialysis Center of King Fahd Specialized Hospital, Buraydah, Qassim Region, Saudi Arabia. The hospital is a tertiary referral facility providing comprehensive renal replacement therapy services to patients with chronic kidney disease across the region.

### Study period

The study was conducted over a 16-month period, encompassing study preparation, data collection, data management, statistical analysis, and manuscript preparation. Participant recruitment and data collection were conducted over four consecutive months.

### Study population and sample size

The study population comprised adult patients undergoing maintenance hemodialysis at the Dialysis Center of King Fahd Specialized Hospital. Eligible participants were adults aged 18 years or older who had been receiving maintenance hemodialysis for at least three months, were clinically stable, able to communicate, and willing to provide written informed consent. Patients with acute dialysis-related complications requiring emergency management, severe cognitive impairment, diagnosed psychiatric disorders, or medical conditions unrelated to chronic kidney disease that could substantially impair physical function (e.g., recent stroke, major trauma, or limb amputation) were excluded.

A non-probability convenience sampling technique was employed, whereby all eligible patients attending the dialysis center during the data collection period were invited to participate. The required sample size was estimated to be *a priori* using G*Power version 3.1.9.7 for multiple regression analysis. Assuming a medium effect size (f² = 0.15), a significance level (α) of 0.05, a statistical power of 80%, and ten independent predictor variables, the minimum required sample size was estimated at 118 participants. An additional 12 participants (approximately 10% of the calculated sample) were recruited for the pilot study, resulting in a total of 130 eligible patients approaching. Of the 130 eligible patients, five declined to participate. Among the 125 patients who provided informed consent, 12 participated in the pilot study and were excluded from the final analysis. The remaining 113 participants were enrolled in the main study, of whom three subsequently withdrew, resulting in a final analytical sample of 110 participants. The participant recruitment and inclusion process is illustrated in (**Figure 2**).

**Figure 2:**
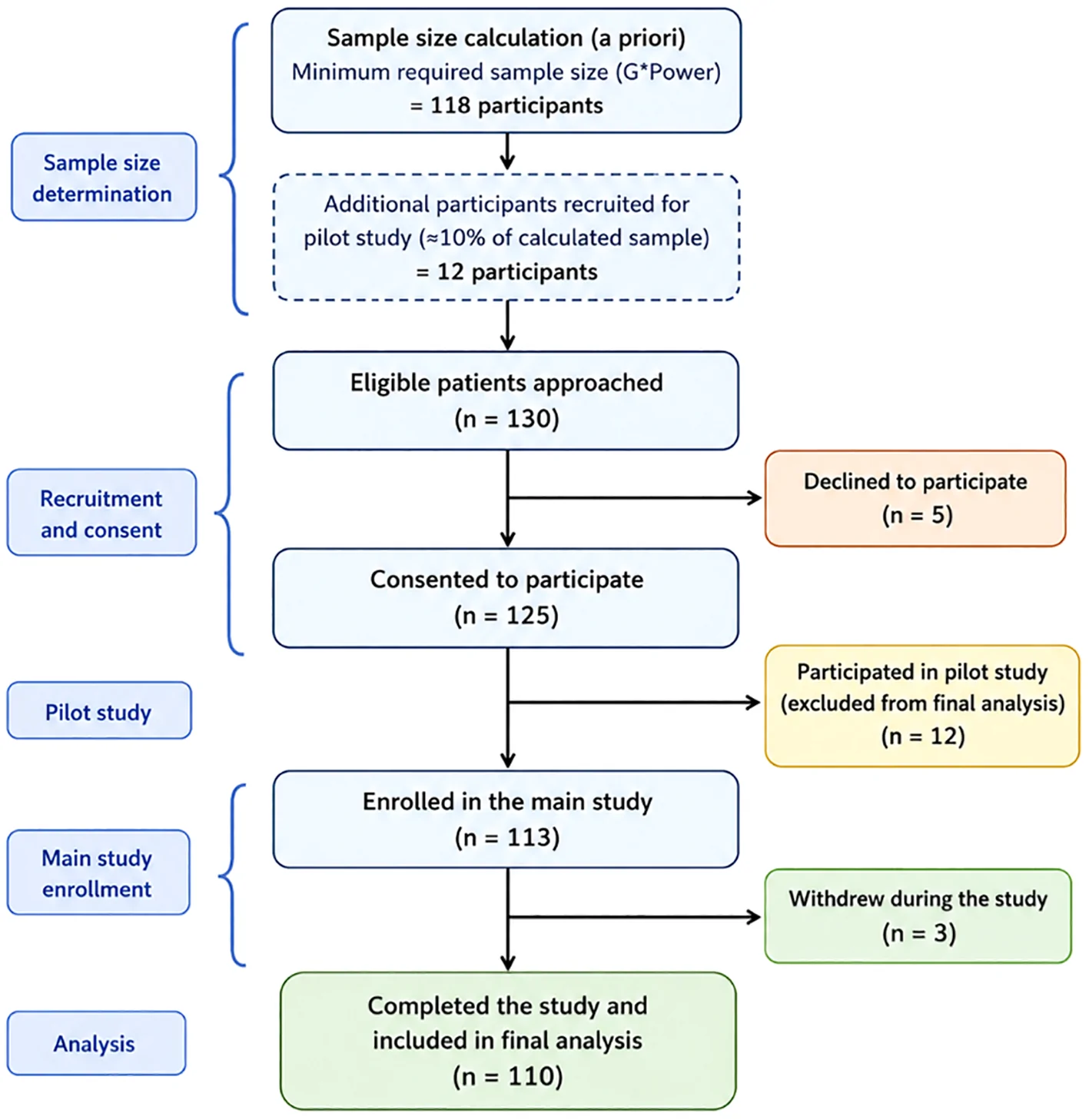
Flow diagram of participant recruitment and inclusion in the study. An additional 12 participants (approximately 10% of the calculated sample size) were recruited for the pilot study. Pilot study participants were excluded from the final analysis to prevent potential bias.

### Data collection tools

Data were collected using a structured electronic questionnaire developed in Google Forms and distributed to participants via a Quick Response (QR) code and a secure web link during their scheduled hemodialysis sessions. The questionnaire was adapted from previously validated instruments, translated from English into Arabic using a standardized forward–backward translation procedure, and reviewed by experts to ensure conceptual equivalence, linguistic accuracy, and cultural appropriateness before administration. Data consisted of three sections.

The first section, adapted from Abd El Mawgod et al. (16), included 15 items assessing participants’ sociodemographic and clinical characteristics, including age, gender, educational level, marital status, cause of end-stage renal disease, duration of hemodialysis, duration of dialysis sessions, and the presence of comorbidities, which were recorded as a dichotomous variable (yes or no); specific types of comorbid conditions were not systematically collected.

The second section comprised a 40-item Physical Function Questionnaire adapted from Milenkova et al. (8). This instrument assessed participants’ physical function and their ability to perform activities of daily living, including mobility, self-care, household activities, and physical activities. Activities of daily living were scored using a four-point response scale reflecting the participant’s level of independence: 0 = unable to perform the activity, 1 = performs the activity with complete assistance, 2 = performs the activity independently, and 3 = not applicable. Items assessing sleep-related problems were rated on a five-point Likert scale ranging from 0 (rarely) to 5 (all the time). A 50-point threshold was used for classification based on the scoring criterion referenced by Wilson et al. (17), with scores <50 classified as low physical function and scores ≥50 as high physical function.

The third section, adapted from Abdel Kareem et al. (18), consisted of 26 items evaluating clinical characteristics and dialysis-related health status. These included anthropometric measurements (height and weight), laboratory parameters (serum calcium, iron, ferritin, and albumin levels), and common dialysis-related symptoms and complaints such as edema, headache, anorexia, obesity, and lethargy.

### Validity

The content validity of the study questionnaire was established through evaluation by a panel of five experts in medical-surgical nursing and nephrology nursing. The experts reviewed the instrument for clarity, relevance, comprehensiveness, and cultural appropriateness. Based on their recommendations, minor modifications were made to improve the wording and sequencing of several items before data collection.

### Reliability

The internal consistency of the study instruments was assessed using Cronbach’s alpha coefficient. The Physical Function Questionnaire (Section II) demonstrated good internal consistency, with a Cronbach’s alpha coefficient of 0.85, while the Clinical Characteristics and Dialysis-Related Health Status Questionnaire (Section III) demonstrated acceptable internal consistency, with a Cronbach’s alpha coefficient of 0.81, indicating satisfactory reliability of both instruments.

### Pilot study

A pilot study was conducted among 12 patients (10%) of the target population to evaluate the feasibility, clarity, and applicability of the study instruments and the data collection procedures. Based on the findings and expert feedback, minor modifications were made to improve the clarity of several questionnaire items. Participants included in the pilot study were excluded from the final study sample to prevent potential bias.

### Data collection procedure

Data were collected over a four-month period from October 2025 to January 2026 by investigators and trained research assistants at the Dialysis Center of King Fahd Specialized Hospital, Buraydah, Saudi Arabia. Eligible patients were identified through the dialysis unit records in collaboration with the nursing staff and were approached individually before or during their scheduled hemodialysis sessions. The purpose and procedures of the study were explained to each participant, and those who agreed to participate provided written informed consent prior to data collection. A secure electronic questionnaire was developed using Google Forms, and participants were invited to complete the survey by scanning a Quick Response (QR) code or accessing a secure web link using their personal smartphones or tablet devices during their dialysis session.

Participants who experienced difficulties completing the electronic questionnaire because of advanced age, limited literacy, visual impairment, or physical limitations received standardized assistance from trained members of the research team, who read the questions verbatim and recorded participants’ responses without interpretation or influence. Completion of the questionnaire required approximately 20–30 minutes per participant. Anthropometric measurements and laboratory data were extracted from the participants’ most recent medical records on the day of data collection using a standardized data extraction form after obtaining the necessary institutional approvals. To ensure data quality, all submitted questionnaires were reviewed for completeness and consistency before analysis, and all collected data were anonymized and securely stored to maintain participants’ confidentiality.

### Statistical analysis

Data were analyzed using IBM SPSS Statistics version 20.0 (IBM Corp., Armonk, NY, USA). Descriptive statistics were used to summarize participants’ characteristics and study variables. The normality of continuous variables was assessed using the Shapiro–Wilk test, and all continuous variables presented as mean ± SD demonstrated approximately normal distributions. Continuous variables were presented as means and standard deviations (SD), whereas categorical variables were summarized using frequencies and percentages. Associations between categorical variables were examined using the Chi-square test or Fisher’s exact test, as appropriate. Pearson’s correlation coefficient was used to assess relationships between continuous variables. To identify factors independently associated with impaired physical function, multivariable binary logistic regression analysis was performed, with impaired physical function coded as 1 and preserved physical function as 0. The model included age, sex, body mass index, duration of hemodialysis, hemoglobin level, serum albumin level, and comorbidity status. Results were reported as adjusted odds ratios (AORs) with 95% confidence intervals (CIs). Model fit was assessed using the Hosmer–Lemeshow goodness-of-fit test and Nagelkerke R². All statistical tests were two-tailed, and a p-value <0.05 was considered statistically significant.

## Results

**Table 1** clarify more than half of the participants were male (55.5%), and the mean age was 56.8 ± 14.2 years. The largest proportion of participants was aged >60 years (36.4%), followed by those aged 50–60 years (25.5%) and 35–49 years (22.7%). Most participants were married (74.5%), while 14.5% were single, 9.1% were widowed, and 1.8% were divorced. Regarding educational level, 37.3% could read and write, 32.7% had primary education, 17.3% had secondary education, 10.0% had university education, and 2.7% had postgraduate education. The majority of participants were unemployed (71.8%), whereas 15.5% were employed and 12.7% were retired. Concerning living arrangements, 39.1% lived with family members, 31.8% lived with their spouse, 16.4% resided in care facilities, 5.5% lived with their children, and 7.3% lived alone.

**Table 1:** Sociodemographic characteristics of patients undergoing maintenance hemodialysis (N = 110).

Characteristic	n (%)
Gender	
■ Male	61 (55.5)
■ Female	49 (44.5)
Age group (years)	
■ 18–24	7 (6.4)
■ 25–34	10 (9.1)
■ 35–49	25 (22.7)
■ 50–60	28 (25.5)
■ >60	40 (36.4)
Mean ± SD	56.8 ± 14.2
Marital status	
■ Single	16 (14.5)
■ Married	82 (74.5)
■ Divorced	2 (1.8)
■ Widowed	10 (9.1)
Educational level	
■ Read and write	41 (37.3)
■ Primary education	36 (32.7)
■ Secondary education	19 (17.3)
■ University education	11 (10.0)
■ Postgraduate education	3 (2.7)
Employment status	
■ Unemployed	79 (71.8)
■ Employed	17 (15.5)
■ Retired	14 (12.7)
Living arrangement	
■ Living with spouse	35 (31.8)
■ Living with children	6 (5.5)
■ Living with family	43 (39.1)
■ Residential care facility	18 (16.4)
■ Living alone	8 (7.3)

**Table 2** reveals the mean duration of maintenance hemodialysis was 4.8 ± 2.9 years, with a mean body mass index of 26.7 ± 4.3 kg/m², mean hemoglobin level of 10.4 ± 1.3 g/dL, and mean serum creatinine level of 9.8 ± 2.6 mg/dL. Regarding dialysis duration, 30.9% of participants had been receiving hemodialysis for 2–5 years, 29.1% for <2 years, and 34.5% for >5 years, while 5.5% were unable to recall their dialysis duration. Almost all participants (94.5%) underwent three hemodialysis sessions per week, whereas 3.6% received dialysis twice weekly, and 0.9% each received dialysis once weekly or daily. Physicians were the primary healthcare providers during dialysis for 96.4% of participants, followed by family members (1.8%), nurses (0.9%), and others (0.9%). Nearly (94.5%) of participants had comorbidities, whereas (5.5%) did not. Additionally, 94.5% of participants reported regular attendance at their scheduled hemodialysis sessions, while 5.5% reported irregular attendance.

**Table 2:** Clinical and dialysis-related characteristics of patients undergoing maintenance hemodialysis (N = 110).

Characteristic	Overall (N = 110)
Duration of maintenance hemodialysis (years), mean ± SD*	4.8 ± 2.9
Body mass index (kg/m²), mean ± SD	26.7 ± 4.3
Hemoglobin (g/dL), mean ± SD	10.4 ± 1.3
Serum creatinine (mg/dL), mean ± SD	9.8 ± 2.6
Dialysis duration, n (%)	
■ <2 years	32 (29.1)
■ 2–5 years	34 (30.9)
■ >5 years	38 (34.5)
■ Unable to recall	6 (5.5)
Hemodialysis sessions per week, n (%)	
■ Once	1 (0.9)
■ Twice	4 (3.6)
■ Three times	104 (94.5)
■ Daily	1 (0.9)
Presence of comorbidities, n (%)	
■ Yes	104 (94.5)
■ No	6 (5.5)
Healthcare provider during dialysis, n (%) †	
■ Physician	106 (96.4)
■ Nurse	1 (0.9)
■ Family member	2 (1.8)
■ Other	1 (0.9)
Regular attendance at scheduled dialysis sessions, n (%) ‡	
■ Yes	104 (94.5)
■ No	6 (5.5)

**Table 3** presents most participants (71.8%) were not employed, while 25.5% reported working fewer than 10 hours per week and 2.7% worked 10–20 hours per week. Regarding pain during physical activity, 37.3% reported never experiencing pain, 31.8% always experienced pain, 19.1% sometimes experienced pain, and 11.8% reported often. Concerning pain severity, 37.3% indicated that the item was not applicable, 37.3% reported mild pain, 12.7% moderate pain, 10.0% very mild pain, and 2.7% severe pain. More than half of the participants (52.7%) rated their current health as good, whereas 36.4% rated it as fair, 7.3% as poor, and 1.8% each as excellent or very good. Compared with the previous year, 29.1% perceived their health as better, 18.2% as slightly worse, 20.9% as moderately worse, 27.3% as worse, and 4.5% as severely worse.

**Table 3:** Physical function and self-reported health characteristics of patients undergoing maintenance hemodialysis (N = 110).

Characteristic	n (%)
Weekly working hours	
■ Not employed	79 (71.8)
■ <10 hours	28 (25.5)
■ 10–20 hours	3 (2.7)
■ >20 hours	0 (0.0)
Pain during physical activity	
■ Never	41 (37.3)
■ Sometimes	21 (19.1)
■ Often	13 (11.8)
■ Always	35 (31.8)
Pain severity	
■ Not applicable	41 (37.3)
■ Very mild	11 (10.0)
■ Mild	41 (37.3)
■ Moderate	14 (12.7)
■ Severe	3 (2.7)
Current self-rated health	
■ Excellent	2 (1.8)
■ Very good	2 (1.8)
■ Good	58 (52.7)
■ Fair	40 (36.4)
■ Poor	8 (7.3)
Perceived change in health compared with the previous year	
■ Better	32 (29.1)
■ Slightly worse	20 (18.2)
■ Worse	30 (27.3)
■ Moderately worse	23 (20.9)
■ Severely worse	5 (4.5)

**Table 4** presents the physical function scores and prevalence of impaired physical function among patients undergoing maintenance hemodialysis. Overall, 81 (73.6%) participants had impaired physical function, whereas 29 (26.4%) had preserved physical function. The raw physical function score ranged from 0 to 10 within a possible range of 0–15, with a mean of 5.65 ± 2.17 and a median (IQR) of 6.0 (4.0–8.0). When converted to a percentage of the maximum possible score, the mean physical function score was 37.7 ± 14.5%.

**Table 4:** Physical function scores and prevalence of impaired physical function among patients undergoing maintenance hemodialysis (N = 110).

Physical function assessment	Value
Physical function level, n (%)	
■ Low (<50% of the maximum score)	81 (73.6)
■ High (≥50% of the maximum score)	29 (26.4)
Raw physical function score	
■ Possible score range	0–15
■ Observed score range (Min–Max)	0.0–10.0
■ Mean ± SD	5.65 ± 2.17
■ Median (IQR)	6.0 (4.0–8.0)
Percentage physical function score	
■ Mean percentage score (%) ± SD	37.7 ± 14.5

SD, standard deviation; IQR, interquartile range. Physical function scores were converted to percentages of the maximum possible score; scores <50% (raw score 0–7) were classified as impaired physical function, whereas scores ≥50% (raw score 8–15) were classified as preserved physical function.

**Table 5** summarizes participants’ functional independence in activities of daily living. Most participants performed basic self-care activities independently, including using the toilet (76.4%), dressing (75.5%), walking (65.5%), and bathing (57.3%). Independence was lower for instrumental activities of daily living, with 44.0% of participants shopping independently, 33.6% going to the bank, 22.7% carrying objects, 20.9% washing clothes using a washing machine, 17.3% cooking, 28.2% making the bed or household arrangements, 9.1% cleaning floors, 5.5% washing clothes manually, and 7.3% participating in sports or exercise. Conversely, substantial proportions of participants reported being unable to perform several activities, including participating in sports or exercise (70.9%), washing clothes manually (78.2%), cooking (63.6%), carrying objects (57.3%), washing clothes using a washing machine (60.9%), cleaning floors (61.8%), making the bed or household arrangements (39.1%), shopping (31.8%), and going to the bank (31.8%). The proportions of participants requiring complete or partial assistance varied across activities, while only a small percentage indicated that specific activities were not applicable.

**Table 5:** Functional independence in activities of daily living among patients undergoing maintenance hemodialysis (N = 110).

Activity of daily living	Unable to perform n (%)	Requires complete assistance n (%)	Requires partial assistance n (%)	Independent n (%)	Not applicable n (%)
■ Bathing	8 (7.3)	10 (9.1)	6 (5.5)	83 (75.5)	3 (2.7)
■ Dressing	9 (8.2)	12 (10.9)	3 (2.7)	83 (75.5)	3 (2.7)
■ Using the toilet	8 (7.3)	12 (10.9)	3 (2.7)	84 (76.4)	3 (2.7)
■ Making the bed/household arrangement	43 (39.1)	21 (19.1)	10 (9.1)	31 (28.2)	5 (4.5)
■ Cleaning floors	68 (61.8)	15 (13.6)	12 (10.9)	10 (9.1)	5 (4.5)
■ Washing clothes manually	86 (78.2)	6 (5.5)	8 (7.3)	6 (5.5)	4 (3.6)
■ Washing clothes using a washing machine	67 (60.9)	10 (9.1)	8 (7.3)	23 (20.9)	2 (1.8)
■ Cooking	70 (63.6)	12 (10.9)	6 (5.5)	19 (17.3)	3 (2.7)
■ Going to the bank	35 (31.8)	18 (16.4)	17 (15.5)	37 (33.6)	3 (2.7)
■ Shopping	35 (31.8)	16 (14.5)	12 (10.9)	44 (40.0)	3 (2.7)
■ Participating in sports/exercise	78 (70.9)	6 (5.5)	5 (4.5)	8 (7.3)	13 (11.8)
■ Walking	16 (14.5)	10 (9.1)	8 (7.3)	72 (65.5)	4 (3.6)
■ Carrying objects	63 (57.3)	10 (9.1)	7 (6.4)	25 (22.7)	5 (4.5)

**Table 6** presents the self-reported sleep-related symptoms among patients undergoing maintenance hemodialysis. Regarding sleep quality, 60.9% of participants reported that they rarely slept well, whereas 10.0% reported experiencing sleep problems always, 10.9% often, 6.4% usually, and 4.5% sometimes. Feeling anxious or stressed upon waking during the night was reported always by 60.9% of participants, followed by 24.5% who experienced it usually, 6.4% often, 2.1% sometimes, and 6.4% rarely. Similarly, 56.4% of participants reported always experiencing insomnia or difficulty sleeping, while 27.3% experienced it usually, 8.2% often, 2.7% sometimes, and 5.5% rarely.

**Table 6:** Self-reported sleep-related symptoms among patients undergoing maintenance hemodialysis (N = 110).

Sleep-related symptom	Always n (%)	Often n (%)	Usually n (%)	Sometimes n (%)	Rarely n (%)
■ Sleep well	11 (10.0)	1 (0.9)	26 (23.6)	5 (4.5)	67 (60.9)
■ Wake up at night feeling anxious or stressed	67 (60.9)	7 (6.4)	27 (24.5)	2 (1.8)	7 (6.4)
■ Insomnia or difficulty sleeping	62 (56.4)	9 (8.2)	30 (27.3)	3 (2.7)	6 (5.5)

**Table 7** presents the self-reported post-dialysis symptoms among patients undergoing maintenance hemodialysis. Among the general symptoms, **fatigue** was the most frequently reported (**93.6%**), followed by fever (3.6%). Regarding neurological symptoms, headache was reported by 37.3% of participants, whereas fainting was reported by 5.5%. Gastrointestinal symptoms included nausea in 10.9% of participants and vomiting in 4.5%. Vascular access-related symptoms were less common, with arm swelling reported by 10.9%, inflammation by 8.2%, pain by 6.4%, tenderness by 3.6%, and redness by 2.7% of participants.

**Table 7:** Self-reported post-dialysis symptoms among patients undergoing maintenance hemodialysis (N = 110).

Symptom category	Post-dialysis symptom	n (%)
■ General symptoms	Fatigue	103 (93.6)
	Fever	4 (3.6)
■ Neurological symptoms	Headache	41 (37.3)
	Fainting	6 (5.5)
■ Gastrointestinal symptoms	Nausea	12 (10.9)
	Vomiting	5 (4.5)
■ Vascular access-related symptoms	Arm swelling	12 (10.9)
	Inflammation	9 (8.2)
	Pain	7 (6.4)
	Tenderness	4 (3.6)
	Redness	3 (2.7)

**Table 8** compares nutritional and biochemical parameters according to physical function status, which participants with impaired physical function had a lower mean serum albumin level than those with preserved physical function (39.2 ± 4.9 vs. 41.5 ± 4.5 g/L, respectively; p = 0.030). No statistically significant differences were observed between the two physical function groups regarding hemoglobin,

**Table 8:** Nutritional and biochemical characteristics according to physical function status among patients undergoing maintenance hemodialysis (N = 110).

Laboratory parameter	Unit	Overall (N = 110)	Impaired physical function (n = 81)	Preserved physical function (n = 29)	Test statistic	P-value
■ Hemoglobin	g/dL	10.4 ± 1.3	10.3 ± 1.3	10.7 ± 1.2	t = −1.43	0.156
■ Serum creatinine	mg/dL	9.8 ± 2.6	9.9 ± 2.7	9.5 ± 2.4	t = 0.70	0.486
■ Serum calcium	mg/dL	8.8 ± 0.9	8.7 ± 0.9	9.0 ± 0.8	t = −1.57	0.120
■ Serum iron	µg/dL	63.4 ± 24.7	61.5 ± 24.3	68.7 ± 25.3	t = −1.35	0.180
■ Serum ferritin	ng/mL	1328.2 (781.4–2035.6)	1384.0 (805.0–2110.0)	1185.0 (720.0–1840.0)	U = 1021.0	0.293
■ Serum albumin	g/L	39.8 ± 4.9	39.2 ± 4.9	41.5 ± 4.5	t = −2.20	0.030

Values are presented as mean ± SD or median (IQR), as appropriate. SD, standard deviation; IQR, interquartile range. Independent-samples t-test was used for normally distributed continuous variables, whereas the Mann–Whitney U test was used for non-normally distributed variables. Bold values indicate statistical significance at p < 0.05.

**Table 9** presents a statistically significant association was observed between age group and physical function (χ² = 14.796, p = 0.002). The proportion of participants with impaired physical function increased across the older age groups, with the highest proportion observed among participants aged >60 years (50.6%). No statistically significant associations were found between physical function and employment status (p = 0.662), gender (p = 0.866), marital status (p = 0.114), educational level (p = 0.455), duration of maintenance hemodialysis (p = 0.11**8**), number of hemodialysis sessions per week (p = 0.673), family history of kidney disease (p = 0.891), or the presence of comorbidities (p = 0.380).

**Table 9:** Association between participants’ characteristics and physical function among patients undergoing maintenance hemodialysis (N = 110).

Characteristic	Impaired physical function *(n = 81)* n (%)	Preserved physical function *(n = 29)* n (%)	Test statistic	*p*-value
Employment status				
■ Unemployed	61 (75.3)	18 (62.1)	χ² = 0.825	0.662
■ Employed	12 (14.8)	7 (24.1)		
■ Retired	8 (9.9)	4 (13.8)		
Gender				
■ Male	42 (51.9)	19 (65.5)	χ² = 0.028	0.866
■ Female	39 (48.1)	10 (34.5)		
Age group (years)				
■ 18–24	6 (7.4)	1 (3.4)	χ² = 14.796	0.002
■ 25–34	6 (7.4)	4 (13.8)		
■ 35–49	12 (14.8)	13 (44.8)		
■ 50–60	16 (19.8)	12 (41.4)		
■ >60	41 (50.6)	5 (17.2)		
Marital status				
■ Single	13 (16.0)	3 (10.3)	χ² = 5.391	0.114
■ Married	60 (74.1)	22 (75.9)		
■ Divorced	0 (0.0)	2 (6.9)		
■ Widowed	8 (9.9)	2 (6.9)		
Educational level.				
■ Read and write	30 (37.0)	11 (37.9)	χ² = 3.580	0.455
■ Primary	23 (28.4)	13 (44.8)		
■ Secondary	12 (14.8)	7 (24.1)		
■ University	9 (11.1)	2 (6.9)		
■ Postgraduate	1 (1.2)	2 (6.9)		
Duration of hemodialysis				
■ <2 years	20 (24.7)	12 (41.4)	χ² = 5.545	0.118
■ 2–5 years	26 (32.1)	14 (48.3)		
■ >5 years	29 (35.8)	3 (10.3)		
■ Unable to recall	6 (7.4)	0 (0.0)		
Hemodialysis sessions/week				
■ Once	1 (1.2)	0 (0.0)	Fisher’s exact = 2.469	0.673
■ Twice	4 (4.9)	0 (0.0)		
■ Three	75 (92.6)	29 (100.0)		
■ Daily	1 (1.2)	0 (0.0)		
Family history of kidney disease				
■ Yes	11 (13.6)	4 (13.8)	χ² = 0.232	0.891
■ No	40 (49.4)	13 (44.8)		
■ Don’t know	30 (37.0)	12 (41.4)		
Presence of comorbidities				
■ No	3 (3.7)	3 (10.3)	Fisher’s exact = 0.967	0.380
■ Yes	78 (96.3)	26 (89.7)		

n (%). Pearson’s chi-square (χ²) test was used unless otherwise indicated. Fisher’s exact test was applied when expected cell frequencies were <5. Statistically significant associations are shown in bold (p < 0.05).

**Table 10** presents the multivariable logistic regression analysis of factors associated with impaired physical function among patients undergoing maintenance hemodialysis. After simultaneous adjustment for sex, BMI, duration of hemodialysis, hemoglobin, serum albumin, and comorbidity status, increasing age remained independently associated with impaired physical function (AOR = 1.077, 95% CI: 1.024–1.132; p = 0.004). Each additional year of age was associated with approximately a 7.7% increase in the odds of impaired physical function. No statistically significant independent associations were observed for sex, BMI, duration of hemodialysis, hemoglobin, serum albumin, or comorbidity status (all p > 0.05). The model showed acceptable goodness of fit (Hosmer–Lemeshow p = 0.662) and a Nagelkerke R² of 0.273.

**Table 10:** Multivariable logistic regression analysis of factors associated with impaired physical function among patients undergoing maintenance hemodialysis (N = 110).

Predictor	B	SE	Wald χ²	AOR	95% CI for AOR	P-value
■ Age, years	0.074	0.026	8.10	1.077	1.024–1.132	0.004
■ Sex (male vs. female)	−0.284	0.525	0.29	0.753	0.269–2.107	0.589
■ BMI, kg/m²	0.038	0.048	0.63	1.039	0.945–1.142	0.428
■ Duration of hemodialysis, years	0.113	0.079	2.05	1.120	0.959–1.308	0.152
■ Hemoglobin, g/dL	−0.167	0.164	1.04	0.846	0.613–1.168	0.309
■ Serum albumin, g/L	−0.061	0.052	1.38	0.941	0.850–1.042	0.240
■ Comorbidities (yes vs. no)	0.691	0.823	0.71	1.996	0.398–10.008	0.401
■ Constant	−3.846	3.016	1.63	0.021	—	0.202

AOR, adjusted odds ratio; CI, confidence interval; SE, standard error; BMI, body mass index. Dependent variable: impaired physical function (1 = impaired; 0 = preserved). Reference categories: female sex and absence of comorbidities. Bold values indicate statistical significance at p < 0.05.

## Discussion

The present study determined the prevalence of impaired physical function and examined factors associated with physical function among patients undergoing maintenance hemodialysis in Buraydah, Saudi Arabia. Overall, nearly three-quarters of the participants exhibited impaired physical function, indicating a substantial burden of functional limitation in this population. Participants also reported considerable limitations in instrumental activities of daily living, a high prevalence of post-dialysis fatigue, and frequent sleep-related disturbances. These findings are broadly consistent with international evidence demonstrating reduced physical function and functional capacity among patients receiving maintenance hemodialysis (19, 20). At the regional level, studies among dialysis populations have similarly documented substantial physical and quality-of-life burdens associated with long-term dialysis (7–9). In Saudi Arabia, Almousa et al. (1) reported important quality-of-life challenges among dialysis patients in the Qassim region, providing relevant local context for the functional limitations observed in the present study. Differences in the magnitude of impairment across studies may reflect variations in age distribution, comorbidity burden, dialysis characteristics, measurement approaches, and healthcare settings. Collectively, these findings underscore the importance of routinely assessing physical function as part of comprehensive renal care.

The findings of this study can also be considered in relation to Lazarus and Folkman’s Stress and Coping Theory, which conceptualizes chronic illness as a continuous process of stress appraisal and adaptation (10). Maintenance hemodialysis represents a persistent physiological and psychosocial stressor requiring patients to cope with repeated dialysis sessions, dietary and fluid restrictions, treatment dependence, and multiple chronic symptoms (14). Within this theoretical perspective, functional status may be associated with the balance between illness-related demands and the physical, psychological, and social resources available to patients. However, because the present study employed a cross-sectional design, the observed associations cannot establish temporal or causal relationships. Thus, the functional impairment identified in this study may coexist with multiple disease, treatment, and coping-related factors rather than being interpreted as a direct consequence of any single factor (11).

The participants were predominantly older adults, married, unemployed, and receiving maintenance hemodialysis three times per week. Most had been undergoing dialysis for more than two years and reported one or more comorbid conditions. These characteristics are broadly comparable to those reported by Almousa et al. (2021) among dialysis patients in the Qassim region, providing relevant Saudi and regional context for the present findings (1). Similarly, Doan et al. (2024) reported that patients receiving maintenance dialysis commonly experience multiple physical and psychosocial challenges in the context of advanced age, comorbid conditions, and long-term dialysis therapy (19). The similarities may reflect shared characteristics of maintenance hemodialysis populations, whereas differences across studies may be related to variations in patient demographics, comorbidity profiles, dialysis duration, and healthcare settings.

The laboratory profile showed generally preserved serum albumin levels alongside elevated serum ferritin concentrations. Notably, patients with impaired physical function had lower serum albumin levels than those with preserved physical function, whereas hemoglobin, serum creatinine, calcium, iron, and ferritin did not differ significantly between the two groups. This finding is consistent with Tarca et al. (2020), who highlighted the relevance of nutritional and modifiable physical factors to physical functioning among patients receiving dialysis (20). Lower serum albumin levels may indicate less favorable nutritional or clinical status and may be associated with greater functional vulnerability; however, the cross-sectional design precludes establishing a causal relationship. Meanwhile, elevated ferritin concentrations should be interpreted cautiously, as ferritin may reflect both iron stores and the inflammatory milieu commonly observed in patients receiving maintenance hemodialysis. The absence of significant differences in the other biochemical parameters may reflect differences in clinical characteristics, treatment status, or the relatively modest sample size. Overall, these findings support the importance of incorporating nutritional and biochemical monitoring alongside routine physical function assessment in hemodialysis care.

A principal finding of the present study was that 73.6% of participants had impaired physical function, indicating a substantial burden of functional limitation among patients receiving maintenance hemodialysis. Previous evidence suggests that impaired physical function in this population may be associated with multiple interrelated clinical and behavioral factors, including chronic inflammation, anemia, skeletal muscle wasting, cardiovascular disease, reduced exercise tolerance, fatigue, and physical inactivity. However, these mechanisms were not all directly assessed in the present study and should not be interpreted as causal explanations for the observed impairment. The present findings are consistent with Niu et al. (2021), who reported that lower self-reported physical function was associated with increased risks of hospitalization and all-cause mortality among maintenance hemodialysis patients (21). Similarly, Sutcliffe et al. (2018) reported deterioration in physical function among patients receiving hemodialysis over time (22), while Roshanravan and Patel (2019) emphasized the clinical importance of routine physical function assessment in patients with advanced kidney disease (23). Collectively, these international findings support the substantial functional burden identified in the present Saudi population, although differences in patient age, comorbidity profiles, dialysis characteristics, and methods of physical function assessment may account for variations in the magnitude of impairment across studies. Furthermore, MacKinnon et al. (2018) identified associations between physical function, physical activity, and adverse clinical outcomes in chronic kidney disease populations (24), further supporting the clinical relevance of assessing functional status in this population.

Conversely, some longitudinal studies have suggested that physical decline may not occur uniformly among all patients. Gadaen et al. (2021) observed relatively stable functional outcomes over a two-year follow-up despite progressive arterial stiffness (25). This differs from the substantial functional impairment observed in the present study, although direct comparison should be made cautiously because of differences in study design. Variations in participant characteristics, follow-up duration, dialysis practices, and access to rehabilitation services may also contribute to differences across studies.

The present study demonstrated considerable impairment in instrumental activities of daily living. Most participants reported being unable to perform physically demanding household activities, including cleaning floors, washing clothes manually or using a washing machine, cooking, carrying heavy objects, and participating in sports or exercise. In contrast, most participants remained independent in basic self-care activities such as bathing, dressing, toileting, and walking. This pattern suggests greater limitation in more physically demanding activities while basic self-care abilities remain relatively preserved. These findings are broadly consistent with Tabata et al. (2025), who reported low physical function among older patients with chronic kidney disease and identified several geriatric and clinical factors associated with functional status (26).

Likewise, Junqué Jiménez et al. (2021) reported a significant relationship between lower physical activity levels and poorer functional capacity among patients with advanced chronic kidney disease (27). Together, these findings support an association between reduced physical capacity and limitations in everyday functioning; however, the cross-sectional nature of the present study does not permit conclusions regarding the direction or progression of this relationship. The level of independence observed in the present study also differed from that reported by Wu et al. (2022), who identified poorer physical function among hemodialysis patients with impaired skeletal muscle quality (28). Differences in age distribution, clinical characteristics, dialysis exposure, muscle status, rehabilitation opportunities, and methods used to assess physical function may partly explain the variation between studies.

The multivariable analysis further clarified the factors associated with impaired physical function among patients undergoing maintenance hemodialysis. After adjustment for sex, body mass index, dialysis duration, hemoglobin, serum albumin, and comorbidity status, increasing age remained the only independent factor significantly associated with impaired physical function (AOR = 1.077, 95% CI: 1.024–1.132; p = 0.004), with each additional year of age associated with approximately a 7.7% increase in the odds of impairment. This finding is consistent with previous evidence MacKinnon et al. (2023) & Alqalah et al. (2025) demonstrating an association between advancing age and poorer physical function among patients with kidney disease (24–29). Similarly, studies of dialysis populations by Wu, et al. (2023) & Gemdan et al. (2026) reported lower physical activity and functional capacity among older patients (28–30).

In contrast, sex, body mass index, dialysis duration, hemoglobin, serum albumin, and comorbidity status were not independently associated with impaired physical function in the present study. These findings differ, in part, from previous studies by Kim et al. (2021) & Kofiah et al. (2025) reported associations between physical function and clinical, nutritional, and dialysis-related characteristics (13–31). Such differences may be related to variations in age distribution, comorbidity burden, nutritional and clinical profiles, dialysis characteristics, physical function measures, and sample size across studies. Collectively, the present findings identify advancing age as an important independent marker associated with impaired physical function rather than establishing it as a causal determinant. This finding supports routine functional assessment and consideration of age-sensitive rehabilitation and physical activity strategies, particularly among older patients receiving maintenance hemodialysis.

### The study limitations

Several limitations should be considered when interpreting the findings of this study. First, the cross-sectional design precludes establishing causal relationships, while the single-center setting, convenience sampling, and self-reported assessment of physical function may limit generalizability and introduce recall or reporting bias. Second, comorbidities were assessed as an overall presence/absence variable without detailed information on individual comorbid conditions, precluding assessment of the associations between specific comorbidities and physical function. Third, several potentially relevant determinants of physical function were not directly assessed, including frailty, sarcopenia, muscle wasting, recent unintentional weight loss, physical activity, depression, and inflammatory biomarkers. Sex-by-age interactions and sex-specific factors, including menopausal status, osteoporosis, and age-related muscle loss, were also not evaluated. Detailed family and social support, caregiver assistance, interdialytic weight gain, and objective measures of dialysis adequacy were not assessed. Individual comorbidities, particularly diabetes, were not analyzed separately, and a standardized comorbidity index was not used. Furthermore, the high prevalence of post-dialysis fatigue limited its reliable evaluation as an independent factor associated with impaired physical function, while the absence of information on baseline physical activity and previous occupation limited differentiation between dialysis-related and pre-existing functional limitations. Future multicenter prospective studies with larger and more diverse samples and comprehensive clinical, nutritional, psychosocial, and objective functional assessments are warranted to address these limitations and confirm the present findings.

## Conclusion

This study demonstrated that impaired physical function is prevalent among patients undergoing maintenance hemodialysis in Buraydah, Saudi Arabia, with nearly three-quarters of participants exhibiting impaired physical function. Multivariable analysis identified increasing age as the only independent factor significantly associated with impaired physical function after adjustment for relevant sociodemographic and clinical factors. Participants also experienced considerable limitations in activities of daily living, along with frequent fatigue and sleep-related disturbances. These findings support integrating routine physical function assessment into comprehensive hemodialysis care, with particular attention to older patients. Early identification of functional impairment, individualized nursing interventions, multidisciplinary rehabilitation, and promotion of safe physical activity may help preserve functional independence and improve patient outcomes. Further multicenter longitudinal studies with larger samples are warranted to confirm these associations, explore additional clinical and biochemical determinants, and evaluate interventions aimed at maintaining physical function in this population.

### Study implications

The findings of this study have important implications for nursing practice and public health. Given the high prevalence of impaired physical function among patients undergoing maintenance hemodialysis, routine assessment of physical function should be integrated into standard dialysis care to facilitate the early identification of patients at risk of functional decline. Regular evaluation of activities of daily living, mobility, fatigue, and sleep quality may enable healthcare professionals to implement timely, individualized interventions. From a nursing perspective, multidisciplinary strategies incorporating exercise training, nutritional counseling, fatigue management, sleep hygiene education, and psychosocial support should be integrated into routine hemodialysis care to preserve functional independence and improve patients’ quality of life. In addition, structured rehabilitation programs and patient education initiatives may enhance self-management, promote physical activity, and reduce functional deterioration. At the public health level, these findings support the development of evidence-based policies and comprehensive care models that emphasize functional assessment and rehabilitation as essential components of chronic kidney disease management. Future longitudinal and multicenter studies are warranted to evaluate the effectiveness of targeted interventions in improving physical function and long-term clinical outcomes among patients receiving maintenance hemodialysis.

## Data Availability

The original contributions presented in the study are included in the article/supplementary material. Further inquiries can be directed to the corresponding author.
